# The effect of whole egg consumption on weight and body composition in adults: a systematic review and meta-analysis of clinical trials

**DOI:** 10.1186/s13643-023-02277-3

**Published:** 2023-07-17

**Authors:** Arezoo Sadat Emrani, Sara Beigrezaei, Faezeh Zademohammadi, Amin Salehi-Abargouei

**Affiliations:** 1 Research Center for Food Hygiene and Safety, School of Public Health, Shahid Sadoughi University of Medical Sciences, Yazd, Iran; 2 Department of Nutrition, School of Public Health, Shahid Sadoughi University of Medical Sciences, Yazd, 8915173160 Iran; 3 Yazd Cardiovascular Research Center, Non-communicable Deseases Research Institute, Shahid Sadoughi University of Medical Sciences, Yazd, Iran

**Keywords:** Systematic review, Meta-analysis, Egg, Body weight, Body composition, Body mass index, Waist circumference

## Abstract

**Background:**

A limited number of studies have directly examined the effect of whole eggs on body weight and composition in adults, and they have led to inconsistent results. This study aimed to summarize the evidence on the effect of whole egg consumption on body weight and body composition in adults from clinical trials.

**Methods:**

Online databases were searched from inception to April 2023 for clinical trials that directly or indirectly assessed the effect of whole eggs consumption on anthropometric measures including body weight, body mass index (BMI), waist circumference (WC), and fat-free mass (FFM) in adults. A random effects model was used for meta-analysis.

**Results:**

In total, 32 controlled clinical trials were included in the systematic review. The analyses revealed that whole egg consumption has no significant effect on body weight (*n* = 22), BMI (*n* = 13), WC (*n* = 10), and FFM (*n* = 4, *P* > 0.05). The subgroup analyses showed that whole egg consumption has an increasing effect on body weight and BMI in studies that lasted more than 12 weeks and in unhealthy participants (*P* < 0.05). A significant increasing effect on BMI was found in studies that the control group did not receive any egg (*P* < 0.05). Moreover, in studies that there was no significant difference in energy intake between the intervention and control groups, weight, and WC were significantly increased (*P* < 0.05). Additionally, in studies that participants in the control group received another food or supplement, studies with calorie restriction, and studies on healthy subjects, whole egg intake significantly decreased BMI (*P* < 0.05).

**Conclusions:**

Although whole egg consumption had no adverse effect on body composition and body weight, in overall, it might increase body weight in long term. Egg consumption beneficially affects BMI in healthy people and during weight loss diet.

**Systematic review registration:**

This systematic review and meta-analysis is registered in the International Prospective Register of Systematic Reviews (PROSPERO, Registration number: CRD42022308045).

**Supplementary Information:**

The online version contains supplementary material available at 10.1186/s13643-023-02277-3.

## Background

Obesity as an important global health issue is associated with the risk of several chronic diseases, such as high blood pressure, cardiovascular disease (CVD)s [1], type 2 diabetes (T2D) [2], and osteoarthritis [3]. The worldwide prevalence of overweight and obesity has dramatically increased over the past decades and now approximately one-third of adults are overweight or obese throughout the world [4]. Several factors including genetic and environmental (unhealthy diet, physical inactivity, and air pollution) features are associated with overweight and obesity [5]. Although factors such as education and schooling, changes in technology, and urbanization are effective in the prevalence of obesity [6], but they can almost be prevented by lifestyle changes such as a well-balanced diet and regular physical activity [7]. A Western dietary pattern, which mainly consists of red and processed meat, refined grains, and eggs is associated with increased obesity and overweight [8]. A cohort study suggested that animal protein intake may be positively associated with overweight and obesity, while plant-based protein intake has an inverse association [9]. In addition, studies have shown that snacks, fast foods [10], and sweetened beverages [11] consumption are associated with overweight and obesity. Moreover, high fat intake can lead to greater food intake and weight gain [12]. One of the foods that is high in fat and cholesterol is egg [13].

Eggs are rich in minerals, vitamins, and bioactive compounds and important dietary sources of high-biological value protein, as well [13]. Each large egg is 50 g and provides 78 kcal, 6.29 g protein, and 5.3 g fat, 186 mg of which is cholesterol [14]. Bioactive compounds of egg can have antimicrobial, antioxidant, and anticancer effects [13]. Additionally, researchers have suggested that egg-rich diets can have protective effects against metabolic syndrome (MetS) by increasing HDL levels and reducing inflammation [13, 15]. Moreover, it has been observed that eggs may help weight management due to their high protein content [16]. On the other hand, the high levels of cholesterol content in eggs [13], however, plays a key role in maintaining the structure and function of the brain [17], and it might adversely affect the lipid profile [18]. The association between egg consumption and CVDs has been widely investigated. A meta-analysis found a dose–response positive relation between egg consumption and CVDs [19]. However, Sangah Shin et al. [20] suggested that higher egg consumption may reduce the odds for MetS and all its components. A population-based study performed on Chinese adults showed that egg consumption may improve body fat distribution and it might be beneficial for weight management [21].

Although, few studies [21] have directly examined the association of egg consumption with weight and body distribution, many studies have reported the effect of egg intake on weight and body composition as their secondary outcomes. The results of some studies showed a reducing effect of egg consumption on body weight and composition [22]; however, other studies showed no significant effect on body weight [23].

To the best of our knowledge, to date, limited evidence of reviews conducted on the effect of whole egg consumption on body weight and body composition and the evidence on this issue is conflicting. Therefore, this systematic review and meta-analysis study aimed to summarize the evidence on the effect of whole egg consumption on body weight and body composition.

## Methods

The Preferred Reporting Items for Systematic Reviews and Meta-Analysis (PRISMA) guideline was followed for conducting the current systematic review and meta-analysis [24]. The study protocol was also registered in the international prospective register of systematic reviews (PROSPERO) database (http://www.crd.york.ac.uk/PROSPERO, registration number: CRD42022308045).

### Search strategy

A systematic search was conducted from inception to the 23rd of April 2023 to find related articles on online databases including Scopus, PubMed, and ISI (Web of Science). There was no language or any other restriction. The terms used are presented in Additional file 1. Two authors independently screened the titles and abstracts of the articles inception to the 23th of April 2023 (AE and FZ). Finally, references of the selected articles were checked to detect additional related articles.

### Eligibility criteria

Studies from inception to the 23rd of April 2023 were included in the current systematic review, if (a) examined the effect of whole egg consumption on anthropometric measurements including body weight, body mass index (BMI), waist circumference (WC), fat mass, and fat-free mass (FFM), compared to the control group; (b) were controlled clinical trials in design; and (c) performed in adults (≥ 18 years). Studies were excluded if (a) they did not report outcomes of interest, (b) the intervention period was less than 3 weeks, and (c) conducted on pregnant or lactating women.

### Data extraction

Data extraction was conducted by two researchers independently (AE and FZ), and any disagreement was resolved in consultation with third investigator (ASA). The following data was extracted for each included study: publication details (authors, publication year, geographic region), the characteristics of participants (age, sex, number of subjects in intervention and control groups, the health condition of participants), and study characteristics [design (parallel, randomized, cross-over, or factorial intervention), duration, number of study arms, and the amount of whole egg and comparison food used)]. We extracted the mean and standard deviation (SD) for baseline, change, and post-intervention values for outcome markers.

### Risk of bias assessment

Cochrane Collaboration’s tool for assessing the risk of bias in randomized trials was used to evaluate the quality of eligible studies [25]. The assessment was conducted by one author and a second author double-checked the risk assessment. The following domains were evaluated for each study: (1) randomization process, (2) deviations from the intended interventions (effect of assignment to intervention), (3) deviations from the intended interventions (effect of adhering to intervention), (4) missing outcome data, and (5) measurement of the outcome. Studies were categorized as low risk if all domains were rated as low risk, and some concern if at least one domain was assessed to have some concern and high risk if one or more domains were categorized as high risk. The risk of bias in non-randomized clinical trials was assessed at the outcome level by using the risk of bias in the non-randomized studies of interventions (ROBINS-I) tool [26]. Two authors (AE and SB) performed the quality assessments, and disagreements were resolved by a third author (ASA).

### Statistical analysis

Mean difference and its corresponding standard error (SE) in the change from baseline for body weight, BMI, WC, and FFM between intervention and control groups were calculated and used as effect size for meta-analysis. If the SD for change values were not provided, they were calculated by assuming 0.5 as the correlation coefficient between baseline and end-of-treatment values. In addition, all analyzes were performed with correlation coefficients of 0.2 and 0.8 to make sure that our study was not sensitive to the selected correlation coefficient. Mean values with confidence interval (CI) and also medians, and their interquartile range (IQR) were also converted to mean ± SD using suggested formulas [27].

The overall weighted mean differences and corresponding 95% CIs were calculated by using a random-effects model [28] which takes the between-study heterogeneity into account. Cochrane *Q* test and *I*^*2*^ statistic were used to assess the between studies heterogeneity [29]. To investigate possible sources of heterogeneity, several subgroup analyses based on participants’ health status (healthy, unhealthy with chronic diseases), duration of the intervention (between 3 and 12 weeks/ ≥ 12 weeks), geographic region (USA/other countries), study design (parallel/cross-over), dose of the intervention (< 12 whole eggs per week/ ≥ 12 whole eggs per week), difference in energy intake between the two groups (yes/no/unclear), calorie restriction along with whole egg intervention (yes/no), and the type of food in the control group (no egg/foods except egg) were conducted. To assess the robustness of the overall result, a sensitivity analysis was performed by removing studies from the analyses one by one [30]. Publication bias was checked using Begg’s funnel plots and asymmetry tests (Begg’s test and Egger’s test) [31]. All statistical analyses were performed using STATA version 11.2 (Stata Corp, College Station, TX), and *P* values less than 0.05 were considered as statistically significant.

## Results

### Study selection

**Fig. 1:**
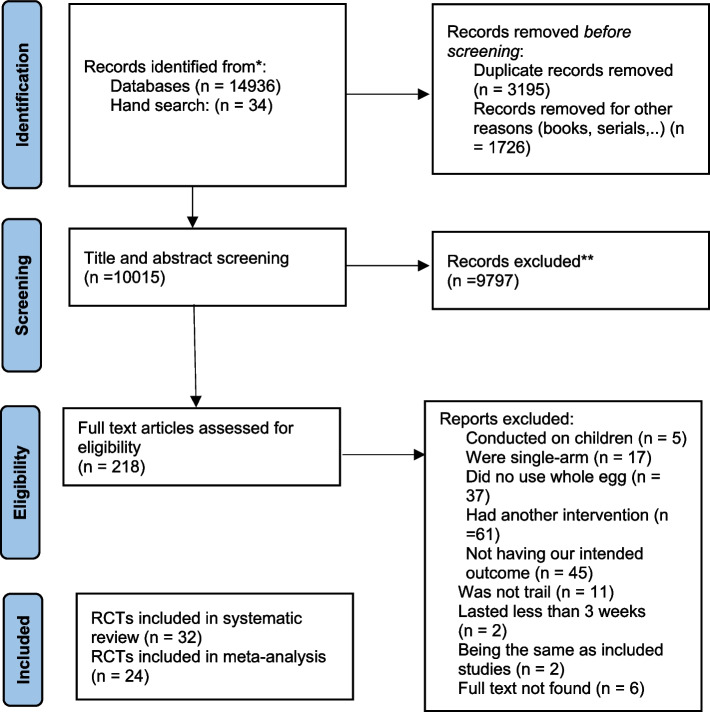
Flow diagram for study selection process

Our systematic search led to 14,970 studies from PubMed, Scopus, Web of Science, and hand search. After removing duplicates and non-article documents (books, series, …), 10,015 studies were screened through reading titles/abstracts. A total of 9797 irrelevant studies were excluded and finally, after reading the full text of 218 potentially relevant studies, 186 studies were excluded because of the following reasons: 5 studies were conducted on children, 17 studies were single-arm, 37 studies did not use the whole egg, 61 studies had another intervention or used enriched eggs for intervention, 11 studies were not the clinical trial in design, 45 studies did not have our intended outcomes, 2 study lasted less than 3 weeks, 2 studies had the same population and protocol as already included studies, and the full text of 6 studies were not available. Therefore, 32 eligible studies were included in our systematic review and meta-analysis [22, 23, 32–61]. The study selection process is provided in Fig. 1.

### Study characteristics

Characteristics of the included studies are shown in Table 1. Eligible studies were published between 1981 and 2022. More than half of the studies were conducted in the USA [22, 23, 32, 34–36, 39, 42, 43, 46, 47, 49, 51, 53, 58, 59, 61]. Five studies were conducted in Australia [37, 38, 40, 44, 60], two in Thailand [45, 52], two in the UK [50, 57], two in India [54, 55], and one in Denmark [56], Pakistan [48], Finland [41], and Mexico [33]. Nineteen studies had a cross-over design [22, 23, 33, 35, 36, 39, 42, 43, 45, 47, 48, 51–57, 61] and 13 were parallel clinical trials [32, 34, 37, 38, 40, 41, 44, 46, 49, 50, 58–60]. All studies were performed on both sexes except two studies one of which was done in females [52], and the other one was conducted in males [59]. Participants in 20 studies were healthy individuals [32, 34, 36, 39–41, 43, 46, 47, 49–51, 53–60]; in five studies, the participants were with T2D or prediabetes [22, 33, 37, 38, 44]; in four studies, they were with MetS [35, 42, 48, 61]; two studies included participants with hyperlipidemia [45, 57]; and participants in a study by Katz et al. [23] were affected by established coronary artery disease (CAD). One of the included studies had two intervention and control groups, as in the intervention groups, one group received two whole eggs per day and the other received two whole eggs per day + 1000 kcal energy deficit diet; therefore, we considered this study as two studies [58]. There were three types of control groups among the studies: most studies excluded whole eggs from the diet of controls [22, 32, 36, 41, 44–50, 52–55, 59, 61] or the control group received fewer whole eggs than intervention group [37, 38, 56, 57, 60]. In eight studies, control groups received a high carbohydrate breakfast without whole egg [23, 33, 34, 39, 40, 42, 43, 58], and one study provided them walnut [51]; and in one study, the control group received choline supplements [35]. Putadechakum et al. [45] reported the values of FFM as a percentage, and in another study, only the overall effect was reported and the difference by diet allocation was not mentioned [44]. So their studies were not included in the meta-analysis for FFM. In the study conducted by Harman et al. [50], only geometric averages were reported, and seven studies not provided data needed for meta-analysis [51–57]; therefore, these studies were only reported in the systematic review.

**Table 1:** Characteristics of clinical trials that were included in systematic review

Authors, publication year	Country	Design	Gender	Duration (weeks)	Health status of participants	Type and amount of intervention	Type and amount of control	Reported data and result
Daly et al. 2022 [60]	Australia	Parallel	Male and female	12	Healthy	Intervention 1: 7 whole eggs/week Intervention 2: 12 whole eggs/week	2 eggs/week	Weight ↔
Njike et al. 2021 [61]	USA	Cross-over	Male and female	6	MetS	2 eggs/week	No egg	Weight ↔ BMI ↔ WC ↔
Keogh et al. 2020 [40]	Australia	Parallel	Male and female	24	Healthy	10 whole eggs/week	breakfast cereal	Weight ↔
Maki et al. 2020 [42]	USA	Cross-over	Male and female	4	MetS/prediabetes	12 whole eggs/week	energy-matched breakfast meals without eggs contained higher-CHO foods	Weight ↔
Marissa DiBella et al. 2020 [35]	USA	Cross-over	Male and female	4	MetS	21 whole eggs/week	choline supplements (choline bitartrate, CB)	Weight ↔ BMI ↔ WC ↔
Shakoor et al. 2020 [48]	Pakistan	Cross-over	Male and female	5	MetS	14 whole eggs/week	No egg	BMI ↔
Aljohi et al. 2019 [32]	USA	Parallel	Male and female	48	Healthy	no more than 2 whole eggs per day, 12 eggs per week and not to go > 2 days without eating eggs	No egg	BMI ↔
Fuller et al. 2018 [37]	Australia	Parallel	Male and female	24	Prediabetes/T2D	12 whole eggs/week	< 2 eggs/week	WC ↔ FFM ↔
Missimer et al. 2017 [43]	USA	Cross-over	Male and female	4	Healthy	12 whole eggs/week	one packet of oatmeal per day	Weight ↔ BMI ↔ WC ↔
DiMarco et al. 2017 [36]	USA	Cross-over	Male and female	4	Healthy	Intervention 1: 7 whole eggs/week Intervention 2: 14 whole eggs/week Intervention 3: 21 whole eggs/week	No egg	BMI ↔ WC ↔
Fuller et al. 2016 [38]	Australia	Parallel	Male and female	12	Prediabetes/T2D	12 whole eggs/week	less than 2 eggs/wk	Weight ↔ WC ↔ FFM ↔
Njike et al. 2016 [22]	USA	Cross-over	Male and female	12	T2D	10–14 whole eggs/week	No egg	Weight ↑ BMI ↑ WC ↔
Clayton et al. 2015 [34]	USA	Parallel	Male and female	12	Healthy	egg-based breakfasts (14 whole eggs/week)	bagel-based breakfasts (9-cm-diameter bagel and the selected options)	Weight ↔ FFM ↔
Katz et al. 2015 [23]	USA	Cross-over	Male and female	6	Established CAD	breakfast with 2 eggs daily (14 whole eggs/week)	a high-carbohydrate breakfast daily	Weight ↔ BMI ↔
Ballesteros et al., 2015 [33]	Mexico	Cross-over	Male and female	5	T2D	7 whole eggs/week	40 g of oatmeal with 2 cups (472 mL) of lactose-free milk/day	Weight ↔ BMI ↔
Bonny Burns-Whitmore et al. 2014 [51]	USA	Cross-over	Male and female	8	Healthy	6 whole eggs/week	walnuts (28.4 g, 6x/week)	Weight ↔
Khosla et al., 2013 [46]	USA	Parallel	Male and female	14	Healthy	At least 10 whole eggs/week	No egg	Weight ↔
Putadechakum et al. 2013 [45]	Thailand	Cross-over	Male and female	4	Hyperlipidemic	Intervention 1: 7 whole eggs/week Intervention 2: 21 whole eggs/week	No egg	Weight ↔ BMI ↔ WC ↔ FFM ↔
Taweesak Techakriengkrai et al. 2012 [52]	Thailand	Cross-over	Female	4	Hypercholesterolemic	21 whole eggs/week	1 egg/day	Weight ↔ BMI ↔
Pearce et al. 2011 [44]	Australia	Parallel	Male and female	12	T2D	high-protein high-cholesterol (14 whole eggs/week)	High-protein low-cholesterol (no eggs with 100 g of lean protein, meat, chicken or fish)	Weight ↔
Vislocky et al. 2009 [49]	USA	Parallel	Male and female	8	Healthy	12 whole eggs/week	No egg	Weight ↔ FFM ↔
Harman et al. 2008 [50]	UK	Parallel	Male and female	12	Healthy	14 whole eggs/week	No egg	Weight ↔
Vander Wal et al. 2008 [58]	USA	Parallel	Male and female	8	Healthy	14 whole eggs/week	Bagel breakfast	BMI ↓ Weight ↓ WC ↔
						14 whole eggs/week + 1000 kcal energy deficit low fat weight loss diet	bagel breakfast + 1000 ca energy deficit low fat weight loss diet	BMI ↓ Weight ↓ WC ↓
Katz et al. 2005 [39]	USA	Cross-over	Male and female	6	Healthy	14 whole eggs/week	60 g uncooked whole oats	BMI ↔
Tannock et al. 2005 [53]	USA	Cross-over	Male and female	4	Healthy	Intervention 1: 14 whole eggs/week Intervention 2: 28 whole eggs/week	No eggs	Weight ↔
Chakrabarty et al. 2004 [54]	India	Cross-over	Male and female	8	Healthy	7 whole eggs/week	No egg	Weight ↔ BMI ↔
Chakrabarty et al. 2002 [55]	India	Cross-over	Male and female	8	Healthy	7 whole eggs/week	No egg	Weight ↔ BMI ↔
Schnohr et al. 1994 [56]	Denmark	Cross-over	Male and female	6	Healthy	14 whole eggs/week	Usual diet	Weight ↔
Lehtimaki et al. 1992 [41]	Finland	Parallel	Male and female	3	Healthy	21 whole eggs/week	No egg	Weight ↔
Edington et al. 1987 [57]	UK	Cross-over	Male and female	8	Group1: healthy Group 2: hyperlipidemia	7 whole eggs/week	2 eggs/ week	Weight ↔
Sacks et al. 1984 [47]	USA	Cross-over	Male and female	3	Healthy	7 whole eggs/week	No egg	Weight ↔
Flaim et al. 1981 [59]	USA	Parallel	Male	5	Healthy	28 whole eggs/week	No egg	Weight ↑

*BMI* Body mass index, *FFM* Fat-free mass, *WC* Waist circumference, *MetS* Metabolic syndrome, *T2D* Type 2 diabetes, *CAD* Cardiovascular disease

^↑^Significant increasing effect, ^↓^significant decreasing effect, ^↔^non-significant effect

### Risk of bias assessment

The results of the risk of bias assessment of included randomized studies are shown in Table 2. Only two studies reported allocation concealment [38, 61]. In one study, whole eggs were given in the form of muffins, so the participants and investigators were blind to the intervention and control groups [47]; however, in other studies, blinding was not possible. The result of the risk of bias assessment for 6 studies was rated as “with some concern” [33, 36, 39, 43, 47, 61] mainly because they did not have any information about their allocation method, 23 studies were high risk [22, 23, 32, 34, 35, 37, 40, 42, 44–46, 48–60] mainly due to lack of information about the blinding of outcome assessors, and one study [38] was rated as low risk of bias. One included non-RCT assessed using the ROBINS-I tool [41]. It was scored as the critical risk of bias (Table 3).

**Table 2:** Study quality and risk of bias assessment using Cochrane risk of bias tool for randomized trials

Author, publication year	Randomization process	Deviations from the intended interventions (effect of assignment to intervention)	Deviations from the intended interventions (effect adhering to intervention)	Missing outcome data	Measurement of the outcome	Selection of the reported result	Overall
Daly et al. 2022 [60]	Some concern	Some concern	low	Low	high	Some concern	high
Njike et al. 2021 [61]	low	Some concern	low	low	low	low	Some concern
Keogh et al. (2020) [40]	Some concern	Some concern	low	low	high	Some concern	High
Maki et al. (2020) [42]	Some concern	Some concern	low	Low	high	Some concern	High
Shakoor et al. (2020) [48]	Some concern	Some concern	low	low	high	Some concern	High
DiBella et al (2020) [35]	Some concern	Some concern	low	low	high	Some concern	High
Aljohi et al. (2019) [32]	Some concern	Some concern	low	low	high	Some concern	High
Fuller et al. (2018) [37]	Some concern	low	low	low	high	Some concern	High
Missimer et al. (2017) [43]	Some concern	Some concern	low	low	low	Some concern	Some concern
Dimarco et al. (2017) [36]	Some concern	Some concern	low	Low	high	Some concern	Some concern
Fuller et al. (2016) [38]	low	low	low	low	low	low	Low
Njike et al. (2016) [22]	Some concern	low	low	low	high	Some concern	High
Clayton et al. (2015) [34]	Some concern	Some concern	low	low	high	low	High
Katz et al. (2015) [23]	Some concern	low	low	low	high	low	High
Ballesteros et al. (2015) [33]	Some concern	Some concern	low	low	low	Some concern	Some concern
Burns-Whitmore et al. (2014) [51]	Some concern	Some concern	Low	Low	High	Some concern	High
Rueda & Pramod Khosla (2013) [46]	Some concern	Some concern	low	low	high	Some concern	High
Putadechakum et al. (2013) [45]	Some concern	Some concern	Low	low	high	Some concern	High
Techakriengkrai et al. (2012) [52]	Some concern	Some concern	Low	Low	High	Some concern	High
Pearce et al. (2011) [44]	Some concern	Some concern	low	Low	high	Some concern	High
Vislocky et al. (2009) [49]	Some concern	Some concern	Low	low	high	Some concern	High
Harman et al. (2008) [50]	Some concern	Some concern	low	low	high	Some concern	High
Vander Wal et al. (2008) [58]	Some concern	Some concern	Low	Low	High	Some concern	High
Katz et al. (2005) [39]	Some concern	Some concern	low	low	low	Some concern	Some concern
Tannock et al. (2005) [53]	Some concern	Some concern	Low	Low	High	Some concern	High
Chakrabarty et al. (2004) [54]	Some concern	Some concern	Low	Low	High	Some concern	High
Chakrabarty et al. (2002) [55]	Some concern	Some concern	Low	Low	High	Some concern	High
Schnohr et al. (1994) [56]	Some concern	Some concern	Low	Low	High	Some concern	High
Edington et al. (1987) [57]	Some concern	Some concern	Low	Low	High	Some concern	High
Sacks et al. (1984) [47]	Some concern	low	low	low	low	low	Some concern
Flaim et al. (1981) [59]	Some concern	Some concern	Low	Low	High	Some concern	High

**Table 3:** Study quality and risk of bias assessment by using the risk of bias in non-randomized studies of interventions (ROBINS-I) tool

Author, publication year	Risk of confounding	Risk of selection bias	Risk of misclassification of interventions	Risk of deviation from intended interventions	Risk of missing data	Risk of misclassification of outcomes	Risk of reporting bias	Overall risk of bias
Lehtimaki et al. 1992 [41]	Critical	Low	Low	Low	Low	No information	Low	Critical

### Findings from the meta-analyses

#### The effect of whole egg consumption on body weight

**Fig. 2:**
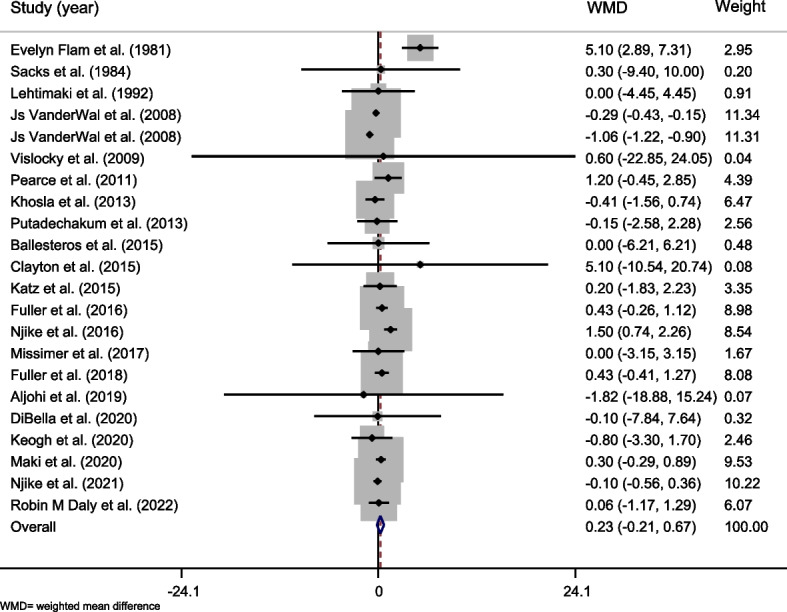
Forest plot representing the effect of egg consumption on body weight using a random-effects model

In total, 21 articles reported data on weight related to whole egg consumption [22, 23, 32–35, 37, 38, 40–47, 49, 58–61]. A total of 1117 subjects participated in these studies. The overall results did not show a significant effect of whole egg consumption on body weight [weighted mean difference (WMD) = 0.234, 95% CI =  − 0.207–0.675, *P* = 0.299) (Table 4 and Fig. 2). A high level of heterogeneity was observed between studies (Cochran *Q* test, *P* < 0.001, *I*^*2*^ = 84.7%). Based on subgroup analyses, results showed a significant positive effect on body weight in studies that lasted 12 weeks or more (WMD = 0.517, 95% CI 0.013–1.021, *P* = 0.044) and studies on unhealthy participants (WMD = 0.452, 95% CI 0.059–0.846, *P* = 0.024. In addition, whole egg consumption had a significant increasing effect on weight in studies that there was no significant difference in energy intakes between the controls and intervention group (WMD = 0.589, 95% CI 0.031–1.147, *P* = 0.039, Table 4).

**Table 4:** The overall effect of whole egg consumption on body weight and by subgroups, using random-effects model

Study group	Number of studies	Number of participants	Meta-analysis			Heterogeneity			
			**WMD**	**95% CI**	***P* effect**	***Q* statistic**	***p* within group**	***I***^***2***^** (%)**	***P* between group**
**Country**									
USA	14	544	0.197	− 0.351, 0.746	0.480	119.24	0.000	89.2%	0.001>
Other countries	8	573	0.371	− 0.077, 0.820	0.105	2.32	0.940	0.0%	
**Study design**									
Parallel	13	802	0.121	− 0.421, 0.663	0.662	98.36	0.000	87.8%	0.001>
Cross-over	9	315	0.409	− 0.140, 0.958	0.145	12.65	0.124	36.8%	
**Control group**									
Nothing	11	616	0.333	− 0.096, 0.763	0.128	14.50	0.151	31.0%	0.001>
Other foods or supplements	11	501	0.130	− 0.494, 0.753	0.684	92.80	0.000	89.2%	
**Duration**									
Less than 12 weeks	13	505	− 0.007	− 0.556, 0.542	0.979	92.11	0.000	87%	0.001>
12 weeks and more	9	612	0.517	0.013, 1.021	0.044	11.55	0.172	30.7%	
**Health status**									
Healthy	12	534	− 0.137	− 0.763, 0.489	0.668	80.13	0.000	86.3%	0.001>
Unhealthy	10	583	0.452	0.059, 0.846	0.024	13.80	0.130	34.8%	
**Dose of intervention**									
Less than 12 eggs per week	4	145	− 0.216	− 1.491, 1.058	0.739	0.04	0.998	0.0%	0.683
12 eggs per week and more	20	1071	0.256	− 0.193, 0.705	0.264	137.45	0.000	86.2%	
**Energy intake differences**									
No	17	823	0.589	0.031, 1.147	0.039	33.76	0.006	52.6%	0.001>
Yes	1	30	0.300	− 0.287, 0.887	0.316	0.00			
Unclear	4	264	− 0.667	− 1.370, 0.037	0.063	52.56	0.000	94.3%	
**Calorie restriction**									
No	18	769	0.367	− 0.107, 0.841	0.129	49.48	0.000	65.6%	0.001>
Yes	4	348	− 0.099	− 1.305, 1.106	0.871	18.44	0.000	83.7%	
**Overall**	22	1117	0.234	− 0.207, 0.675	0.299	136.85	0.000	84.7%	-

*WMD* Weighted mean difference, *CI* Confidence interval

#### The effect of whole egg consumption on BMI

**Fig. 3:**
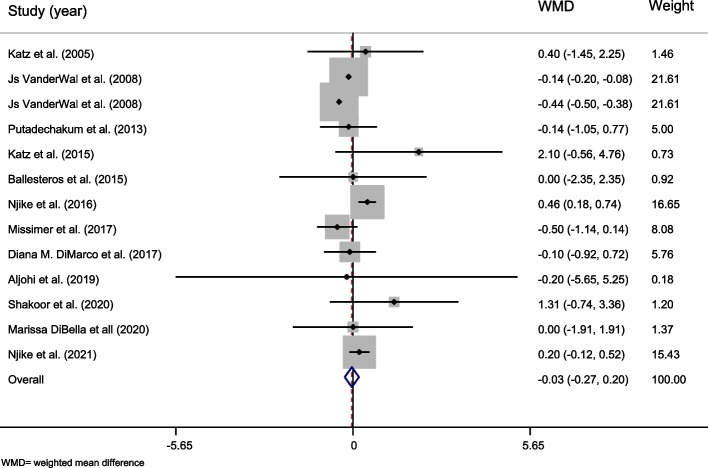
Forest plot representing the effect of egg consumption on body mass index (BMI) using a random-effects model

In total, 12 studies with 13 effect sizes and 570 participants were included in the meta-analysis [22, 23, 32, 33, 35, 36, 39, 43, 45, 48, 58, 61]. The overall effect of whole egg consumption on BMI was not significant (WMD =  − 0.035, 95% CI =  − 0.265–0.196, *P* = 0.769, Table 5 and Fig. 3). The between-study heterogeneity was significant (Cochran *Q* test, *P* = 0.000, *I*^*2*^ = 87%). Subgroup analysis revealed that whole egg consumption significantly increased BMI in studies that excluded whole egg in the diet of controls (WMD = 0.308, 95% CI 0.111–0.505, *P* = 0.002), in studies that lasted 12 weeks and more (WMD = 0.458, 95% CI 0.181–0.735, *P* = 0.001), and in unhealthy participants (WMD = 0.337, 95% CI:0.137 – 0.538, P = 0.001). In contrast, subgroup analysis showed significant decreasing effect of whole egg consumption on BMI in trials that gave controls another food or supplement (WMD − 0.270, 95% CI − 0.532 – − 0.008, *P* = 0.043), in trials with calorie restriction (WMD =  − 0.440, 95% CI − 0.499 – − 0.381, *P* = 0.000) and in healthy individuals (WMD =  − 0.286, 95% CI − 0.538 – − 0.035, *P* = 0.026, Table 5).

**Table 5:** The overall effect of whole egg consumption on body mass index (BMI) and by subgroups, using random-effects model

Study group	Number of studies	Number of participants	Meta-analysis			Heterogeneity			
			**WMD**	**95% CI**	***P* effect**	***Q* statistic**	***P* within group**	***I***^***2***^**%**	***P* between group**
**Country**									
USA	10	450	− 0.046	− 0.287, 0.194	0.707	90.08	0.000	90.0%	0.378
Other countries	3	120	0.087	− 0.694, 0.868	0.828	1.62	0.445	0.0%	
**Study design**									
Parallel	3	197	− 0.290	− 0.580, 0.001	0.051	50.00	0.000	96.0%	0.001>
Cross-over	10	373	0.177	− 0.096, 0.449	0.203	11.92	0.218	24.5%	
**Control group**									
Nothing	6	239	0.308	0.111, 0.505	0.002	4.43	0.490	0.0%	0.001>
Other foods or supplement	7	331	− 0.270	− 0.532, − 0.008	0.044	54.19	0.000	88.9%	
**Duration**									
Less than 12 weeks	11	493	− 0.148	− 0.374, 0.079	0.201	65.67	0.000	84.8%	0.001>
12 weeks and more	2	77	0.458	0.181, 0.735	0.001	0.06	0.813	0.0%	
**Health status**									
Healthy	6	330	− 0.286	− 0.538, − 0.035	0.026	51.15	0.000	90.2%	0.001>
Unhealthy	7	240	0.337	0.137, 0.538	0.001	5.28	0.508	0.0%	
**Dose of intervention**									
Less than 12 eggs per week	3	136	− 0.047	− 0.637, 0.544	0.877	0.03	0.984	0.0%	0.471
12 eggs per week and more	12	541	− 0.036	− 0.269, 0.197	0.762	92.40	0.000	88.1%	
**Energy intake differences**									
No	9	349	0.150	− 0.128, 0.428	0.290	10.87	0.209	26.4%	0.001>
Yes	0	-	-	-	-	-	-	-	
Unclear	4	221	− 0.243	− 0.533, 0.047	0.100	52.88	0.000	94.3%	
**Calorie restriction**									
No	12	491	0.079	− 0.199, 0.356	0.579	27.06	0.005	59.3%	0.001>
Yes	1	79	− 0.440	− 0.499, − 0.381	0.000	0.00			
**Overall**	13	570	− 0.035	− 0.265, 0.196	0.769	92.48	0.000	87.0%	-

*WMD* Weighted mean difference, *CI* Confidence interval

#### The effect of whole egg consumption on waist circumference

**Fig. 4:**
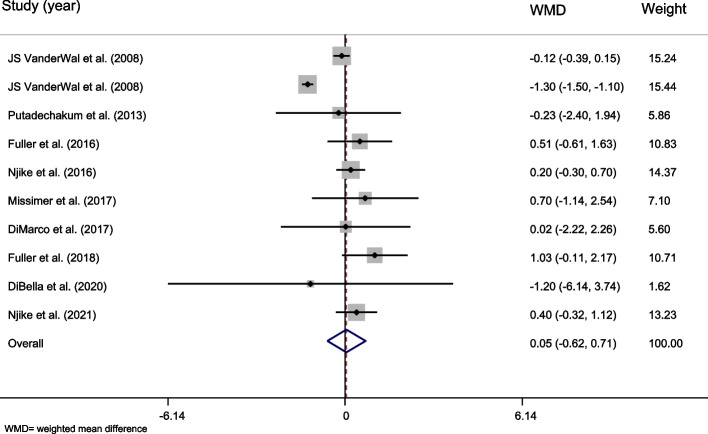
Forest plot representing the effect of egg consumption on waist circumference (WC) using a random-effects model

Ten effect sizes from 9 studies [22, 35–38, 43, 45, 58, 61] (*n* = 665) reported the effect of whole egg consumption on WC (Table 6). The overall effect was not significant (WMD = 0.046, 95% CI =  − 0.616–0.709, *P* = 0.891, Fig. 4). The overall heterogeneity was high (Cochran *Q* test, *P* = 0.000, *I*^*2*^ = 90.1%). In subgroup analysis, a significant effect of egg consumption on WC was observed in trials with no significant difference in energy intake between the intervention and control groups (WMD = 0.350, 95% CI = 0.003–0.698, *P* = 0.048, Table 6).

**Table 6:** The overall effect of whole egg consumption on waist circumference (WC) and by subgroups, using random-effects model

Study group	Number of studies	Number of participants	Meta-analysis			Heterogeneity			
			**WMD**	**95% CI**	***P* effect**	***Q* statistic**	***P* within group**	***I***^***2***^**%**	***P* between group**
**Country**									
USA	7	326	− 0.149	− 0.910, 0.612	0.702	77.11	0.000	92.2%	0.001>
Other countries	3	339	0.647	− 0.101, 1.395	0.090	1.12	0.570	0.0%	
**Study design**									
Parallel	4	420	− 0.094	− 1.043, 0.855	0.846	63.61	0.000	95.3%	0.001>
Cross-over	6	245	0.253	− 0.134, 0.639	0.200	1.00	0.963	0.0%	
**Control group**									
Nothing	6	442	0.345	− 0.010, 0.700	0.057	2.18	0.824	0.0%	0.001>
Other food or supplement	4	223	− 0.486	− 1.507, 0.535	0.351	49.95	0.000	94.0%	
**Duration**									
Less than 12 weeks	7	365	− 0.236	− 1.043, 0.571	0.567	62.64	0.000	90.4%	0.001>
12 weeks and more	3	300	0.360	− 0.064, 0.783	0.096	1.80	0.407	0.0%	
**Health status**									
Healthy	4	236	− 0.398	− 1.372, 0.576	0.423	50.56	0.000	94.1%	0.001>
Unhealthy	6	429	0.345	− 0.013, 0.704	0.059	2.47	0.781	0.0%	
**Dose of intervention**									
Less than 12 eggs per week	2	107	− 0.077	− 1.624, 1.471	0.923	0.01	0.924	0.0%	0.444
12 eggs per week and more	10	665	0.049	− 0.614, 0.712	0.885	90.97	0.000	90.1%	
**Energy intake differences**									
No	8	513	0.350	0.003, 0.698	0.048	2.69	0.912	0.0%	0.001>
Yes	-	-	-	-	-	-	-	-	
Unclear	2	152	− 0.714	− 1.870, 0.442	0.226	47.04	0.000	97.9%	
**Calorie restriction**									
No	8	458	0.024	− 0.195, 0.244	0.827	4.13	0.765	0.0%	0.001>
Yes	2	207	− 0.205	− 2.484, 2.074	0.860	15.67	0.000	93.6%	
**Overall**	10	665	0.046	− 0.616, 0.709	0.891	90.87	0.000	90.1%	-

*WMD* Weighted mean difference, *CI* Confidence interval

#### The effect of whole egg consumption on fat-free mass

**Fig. 5:**
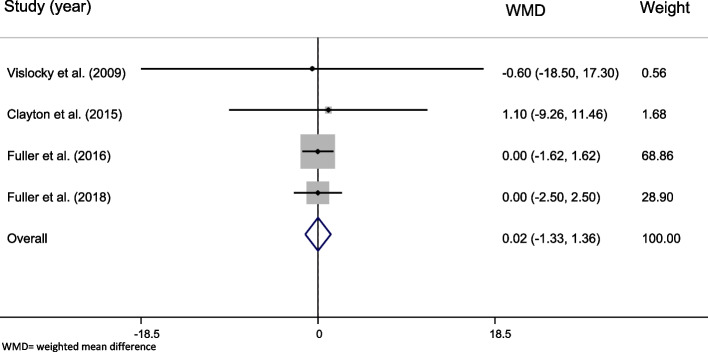
Forest plot representing the effect of egg consumption on body fat-free mass (FFM) using a random-effects model

The effect sizes obtained from four studies [34, 37, 38, 49] with 304 participants did not show any significant effect of whole egg consumption on FFM (WMD = 0.015, 95% CI =  − 1.328–1.358, *P* = 0.982, Fig. 5). No significant effect was found in subgroup analyses (Table 7).

**Table 7:** The overall effect of whole egg consumption on fat free mass (FFM) and by subgroups, using random-effects model

Study group	Number of studies	Number of participants	Meta-analysis			Heterogeneity			
			**WMD**	**95% CI**	***P* effect**	***Q* statistic**	***P* within group**	***I***^***2***^**%**	***P* between group**
**Country**									
USA	2	36	0.673	− 8.292, 9.639	0.883	0.03	0.872	0.0%	0.884
Other countries	2	268	0.000	− 1.359, 1.359	1.000	0.00	1.000	0.0%	
**Duration**									
Less than 12 weeks	1	11	− 0.600	− 18.498, 17.298	0.948	0.00			0.884
12 weeks and more	3	293	0.019	− 1.329, 1.366	0.978	0.04	0.979	0.0%	
**Health status**									
Healthy	2	36	0.673	− 8.292, 9.639	0.883	0.03	0.872	0.0%	0.946
Unhealthy	2	268	0.000	− 1.359, 1.359	1.000	0.00	1.000	0.0%	
**Overall**	4	304	0.015	− 1.328, 1.358	0.982	0.05	0.997	0.0%	-

*WMD* Weighted mean difference, *CI* Confidence interval

#### Publication bias and sensitivity analysis

**Fig. 6:**
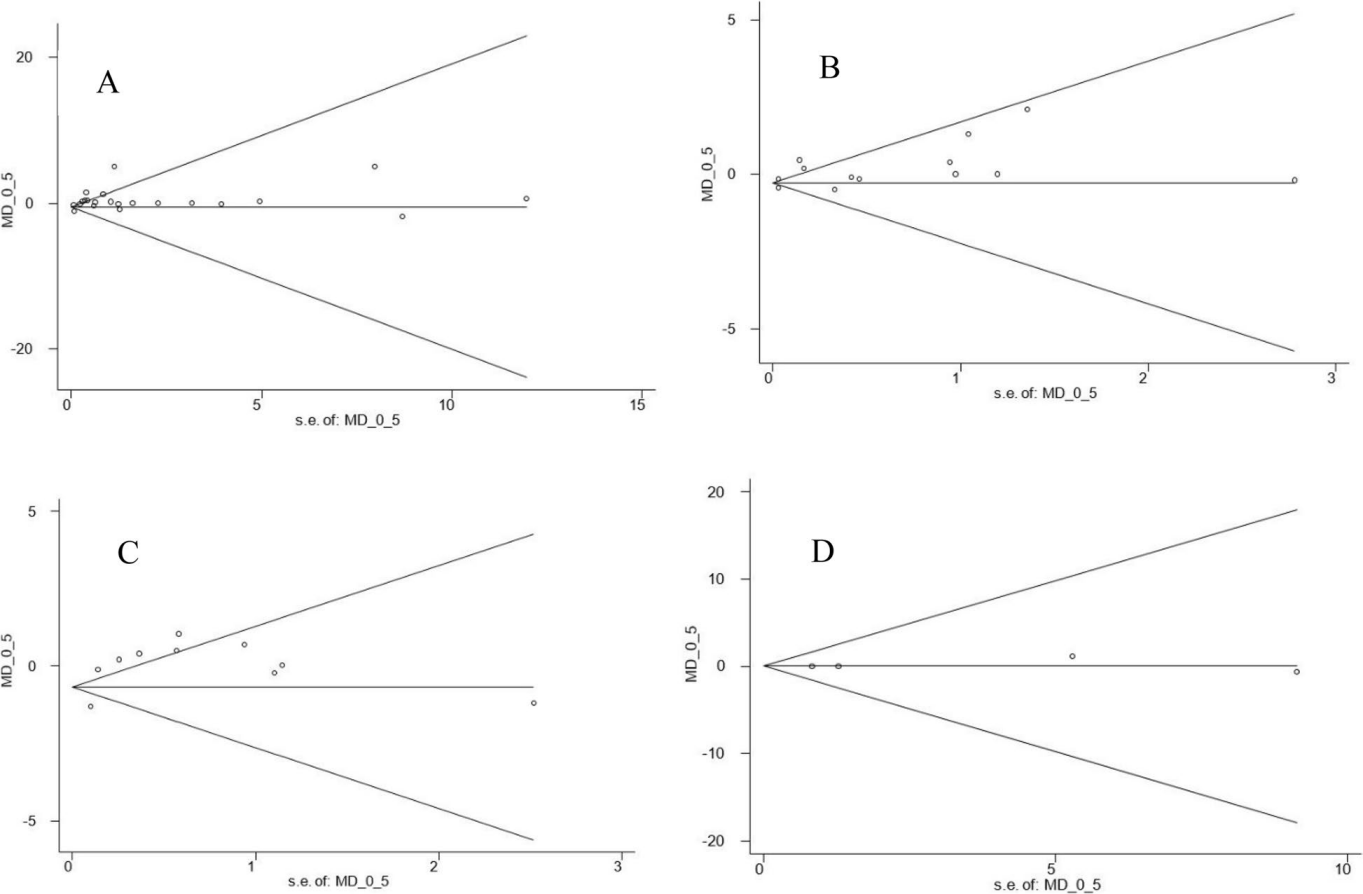
Begg’s funnel plots presenting the effect sizes versus their standard errors (SEs) for controlled trials that assessed the effect of whole egg consumption on weight (**A**), body mass index (BMI) (**B**), waist circumference (WC) (**C**), and fat free mass (FFM) (**D**)

Sensitivity analysis did not show any tangible changes after removing the studies one by one from the meta-analyses. Also, no publication bias was observed for the meta-analysis of BMI (Begg’s test, *P* = 0.463; Egger’s test, *P* = 0.222), WC (Begg’s test, *P* = 0.05; Egger’s test, *P* = 0.1) and FFM (Begg’s test, *P* = 1.000; Egger’s test, *P* = 0.599). However, Begg’s test for meta-analysis of body weight was shown to be significant (Begg’s test, *P* = 0.006; Egger’s test, *P* = 0.046) using statistical asymmetry tests (Fig. 6). Therefore, a trim and fill analysis was used to see if correcting the asymmetry by imputing studies changes the overall effects. The analysis could not add studies and the overall effect was not changed (WMD = 0.234, 95% CI =  − 0.207–0.675, *P* = 0.299).

## Discussion

The current systematic review and meta-analysis of clinical trials were conducted on the effect of whole egg consumption on body weight and body composition in adults. We did not find a significant effect of whole egg consumption on body weight, BMI, WC, and FFM in adults. However, subgroup analyses revealed that whole egg consumption might significantly increase body weight and BMI in studies longer than 12 weeks and in unhealthy subjects. In addition, an increasing effect of whole egg consumption on BMI was observed in studies that the control group did not receive any food as a replacement for egg. In trials with no significant difference in energy intake between the intervention and control groups, a significant increasing effect of egg consumption on weight and WC was found. Furthermore, in trials that the control groups received another food or supplement, in studies with calorie restriction and in healthy participants, a significant decrease was observed in BMI.

Most studies have considered eggs as an important source of cholesterol and have linked them to chronic diseases, especially CVDs and diabetes [62, 63]. But eggs also are rich in high-quality protein, phospholipids, and antioxidants [13] that can have beneficial effects.

A limited number of human studies have directly examined the effect of whole egg consumption on body weight. A study on rats demonstrated that whole egg-based diet may reduce body weight gain and visceral fat in rats [64]. A randomized clinical trial that investigated the effect of egg intake on appetite found that eating an egg-based breakfast could reduce appetite and short-term energy intake [65]. Evidence suggests that eggs may control appetite and increase satiety by inhibiting or stimulating certain hormones like anorexigenic hormones, such as peptide YY (PYY) and glucagon-like peptide-1 (GLP-1) [66]. Contrary to these results, the present systematic review and meta-analysis showed that whole egg intake does not lead to weight loss but also has no effect on weight gain. The reason for this inconsistency may be the short duration of the previous studies, because our systematic review in subgroup analysis revealed that when whole egg is consumed for 12 weeks or more, it can increase body weight and BMI. And also our meta-analysis showed that when there is no difference in energy intake, egg consumption can significantly increase weight. Therefore, in the case of routine and long-term consumption of egg, it is better to take its calorie content into account in the diet. Additionally, we found that whole egg intake has an increasing effect on weight and BMI in unhealthy individuals. However, there is no clear mechanism for this increasing effect, it seems people with diseases such as T2D and CVDs should take whole eggs with caution.

This meta-analysis shows a decreasing effect of whole egg consumption on BMI when there was calorie restriction. The study findings also revealed that BMI increased significantly in studies that excluded eggs from the diet of controls. Previous studies have examined the relationship between egg intake and BMI. Guangzhou Biobank Cohort Study and meta-analysis, a prospective study in 2019, has investigated the association between egg consumption, CVD mortality, and a meta-analysis has reported a negative association between egg consumption and BMI after adjusting by some confounder variables [67]. As mentioned, evidence has shown that egg consumption can increase short-term satiety and fullness and possibly also be effective in controlling weight and reducing BMI. In addition, a clinical trial that examined the effect of an egg breakfast compared to a bagel breakfast on weight loss found that eggs consumed for breakfast can lead to weight loss and decreasing BMI when it is combined with an energy-restricted diet [58] which supports our findings. Although the mechanism involved is not well understood, the extra protein content of egg may contribute to this effect [58].

In our study, egg consumption had no significant effect on WC. Similar to our results, in previous cohort studies and meta-analyses, no association was found between egg consumption and WC [67]. However, in the subgroup analysis, we found an increasing effect of egg intake on WC in the studies that had no difference in energy intake between the intervention and control groups.

In the results of our study, no significant effect was also found on FFM. A recent study that examined the relationship between egg consumption, serum cholesterol levels, and body composition distribution in Korean adult, found no significant association between egg consumption and FFM which support our results [68].

Our study is the first systematic review and meta-analysis that examined the effect of whole egg intake on body weight and composition. It can also be mentioned that a complete and unrestricted search inception to the 23rd of April 2023 was performed for this study. The present study had also some limitations that should be noted. First, heterogeneity between studies were significant. Second, although the magnitude was not high a significant publication bias was observed for the meta-analysis of body weight. Third, only one study had low risks of bias and the majority of included studies had some concerns due to the lack of information about outcome assessors blinding and method of allocation. Moreover, in most included studies, the method of cooking eggs was not specified. As different cooking methods can lead to different effects of food on weight and body composition, it is recommended that future studies consider this issue. It is also important to note that weight and anthropometry measurements were not the primary outcome of most of the included studies, and these studies may not be accurate enough for reporting these secondary outcomes. The studies included in the present meta-analyses were conducted on adults, so the results of this systematic review and meta-analyses cannot be generalized to other age groups. In addition, a number of studies did not provide the needed data for calculating effect sizes [50–57]. The results of these studies were in line with the current findings. Therefore, the overall findings might not change if they were included in the meta-analyses.

## Conclusion

In conclusion, the result of the present systematic review and meta-analysis indicates that whole egg intake might not significantly affect body weight and body composition. However, egg consumption might have adverse effects on body weight and BMI if highly consumed over a long-term period and if consumed by adults with chronic diseases. This review findings also revealed that whole egg might result in better weight reduction if consumed in the context of an energy-restricted diet and if consumed by healthy individuals. Studies that have directly investigated the effect of whole egg consumption on body weight and composition are still lacking, and the mechanisms of this effect have not yet been properly elucidated. Therefore, strong clinical trial studies are needed to measure the effect of whole egg consumption, especially the long-term effect, on weight and anthropometric indices. 

## Supplementary Information

**Additional file 1:** **Supplementary table 1.** Search strategy used for each online database.

## Data Availability

Not applicable.

## Abbreviations

CVD: Cardiovascular disease
T2D: Type 2 diabetes
PRISMA: Preferred Reporting Items for Systematic Reviews and Meta-Analysis
BMI: Body mass index
WC: Waist circumference
FFM: Fat free mass
SD: Standard deviation
SE: Standard error
CAD: Coronary artery disease
WMD: Weighted mean difference
CI: Confidence interval
IQR: Interquartile range
PYY: Peptide YY
GLP-1: Glucagon-like peptide-1
