# Supplementary material for: The effect of whole egg consumption on weight and body composition in adults: a systematic review and meta-analysis of clinical trials
Source: Syst Rev. 2023 Jul 17;12:125. doi: 10.1186/s13643-023-02277-3 (PMC10353215; doi:10.1186/s13643-023-02277-3)
Supplement: Supplementary file 1 — Additional file 1: Supplementary table 1. Search strategy used for each online database. [file 13643_2023_2277_MOESM1_ESM.docx]

| Supplementary table 1. Search strategy used for each online database |
| --- |
| PubMed |
| (("Eggs"[Mesh] OR Egg[tiab] OR Eggs[tiab]) AND (intervention [tiab] OR Intervention*[tiab] OR trial[tiab] OR randomized[tiab] OR blind*[tiab] OR randomised[tiab] OR random[tiab] OR randomly[tiab] OR placebo[tiab] OR assignment[tiab] OR "clinical trial"[publication type] OR RCT[tiab] OR "Clinical Trials as Topic"[Mesh] OR "cross over"[tiab] OR cross-over[tiab] OR parallel[tiab] OR “Body weight”[tiab] OR” body weight”[mesh] OR “Body mass index”[mesh] OR “Body mass index”[tiab] OR BMI[tiab] OR “Waist Circumference”[tiab] OR “Waist Circumference”[mesh] OR WC[tiab] OR WHR[tiab] OR FFM[tiab] OR LBM[tiab] OR FM[tiab] OR "Waist-Hip Ratio"[Mesh] OR "Waist-Hip Ratio"[tiab] OR “lean body mass”[tiab] OR “fat free mass”[tiab])) NOT ("rodentia"[MeSH Terms] OR mouse[tiab] OR mice[tiab] OR rat[tiab] OR pig[tiab] OR rabbit*[tiab] OR rooster[tiab] OR roosters[tiab] OR cell[tiab] OR cells[tiab] OR cow[tiab] OR cows[tiab] OR rabbits[tiab] OR pigs[tiab] OR hamsters[tiab] OR “monkey”[tiab] OR monkeys[tiab] OR goats[tiab] OR shrimp[tiab] OR sheep[tiab] OR cats[tiab] OR dogs[tiab] OR crab[tiab] OR fowl[tiab] OR fowls[tiab] OR roots[tiab] OR bacteria[tiab] OR camel[tiab] OR "cross sectional"[tiab] OR "case control"[tiab] OR "cross-sectional"[tiab] OR "case-control"[tiab] OR "Cohort Studies"[tiab] OR “cohort study”[tiab] OR allergy[tiab] OR allergies[tiab] OR duck[tiab] OR "Parasites"[Mesh] OR malaria[tiab] OR parasite*[tiab] OR egg-laying[tiab] OR "Reproductive Techniques"[Mesh] OR "reproduction"[tiab] OR reproductive[tiab] OR cell*[tiab] OR vaccine[tiab] OR allergen[tiab] OR parasitic[tiab] OR vaccination[tiab] OR immunology[tiab] OR immunization[tiab] OR immunise[tiab] OR immunize[tiab] OR "animal models"[tiab] OR “egg yolk agar”[tiab] OR insect*[tiab] OR "Insecta"[Mesh] OR hen[title] OR quail*[title] OR hens[title] OR chicken*[title] OR broiler*[tiab] OR "Plants"[Mesh] OR “in vitro”[tiab] OR “in-vitro”[tiab] OR rats[tiab] OR “laying”[tiab] OR breed*[tiab] OR frog*[tiab] OR worm[tiab] OR fruitworm[tiab] OR semen[tiab]) |
| Scopus |
| ( ( TITLE-ABS-KEY ( egg )  OR  TITLE-ABS-KEY ( eggs ) ) )  AND  ( ( TITLE-ABS-KEY ( intervention )  OR  TITLE-ABS-KEY ( intervention* )  OR  TITLE-ABS-KEY ( trial )  OR  TITLE-ABS-KEY ( randomized )  OR  TITLE-ABS-KEY ( blind* )  OR  TITLE-ABS-KEY ( randomised )  OR  TITLE-ABS-KEY ( random )  OR  TITLE-ABS-KEY ( randomly )  OR  TITLE-ABS-KEY ( placebo )  OR  TITLE-ABS-KEY ( assignment )  OR  TITLE-ABS-KEY ( rct )  OR  TITLE-ABS-KEY ( "cross over" )  OR  TITLE-ABS-KEY ( "cross-over" )  OR  TITLE-ABS-KEY ( parallel )  OR  TITLE-ABS-KEY ( "body weight" )  OR  TITLE-ABS-KEY ( "body mass index" )  OR  TITLE-ABS-KEY ( bmi )  OR  TITLE-ABS-KEY ( "Waist Circumference" )  OR  TITLE-ABS-KEY ( wc )  OR  TITLE-ABS-KEY ( whr )  OR  TITLE-ABS-KEY ( ffm )  OR  TITLE-ABS-KEY ( lbm )  OR  TITLE-ABS-KEY ( fm )  OR  TITLE-ABS-KEY ( "Waist-Hip Ratio" )  OR  TITLE-ABS-KEY ( "lean body mass" )  OR  TITLE-ABS-KEY ( "fat free mass" ) ) )  AND NOT  ( ( TITLE-ABS-KEY ( mouse )  OR  TITLE-ABS-KEY ( mice )  OR  TITLE-ABS-KEY ( rat )  OR  TITLE-ABS-KEY ( pig )  OR  TITLE-ABS-KEY ( rabbit* )  OR  TITLE-ABS-KEY ( rooster )  OR  TITLE-ABS-KEY ( roosters )  OR  TITLE-ABS-KEY ( cell )  OR  TITLE-ABS-KEY ( cells )  OR  TITLE-ABS-KEY ( cow )  OR  TITLE-ABS-KEY ( cows )  OR  TITLE-ABS-KEY ( rabbits )  OR  TITLE-ABS-KEY ( pigs )  OR  TITLE-ABS-KEY ( hamsters )  OR  TITLE-ABS-KEY ( monkey )  OR  TITLE-ABS-KEY ( monkeys )  OR  TITLE-ABS-KEY ( goats )  OR  TITLE-ABS-KEY ( shrimp )  OR  TITLE-ABS-KEY ( sheep )  OR  TITLE-ABS-KEY ( cats )  OR  TITLE-ABS-KEY ( dogs )  OR  TITLE-ABS-KEY ( crab )  OR  TITLE-ABS-KEY ( fowl )  OR  TITLE-ABS-KEY ( fowls )  OR  TITLE-ABS-KEY ( roots )  OR  TITLE-ABS-KEY ( bacteria )  OR  TITLE-ABS-KEY ( camel )  OR  TITLE-ABS-KEY ( "cross sectional" )  OR  TITLE-ABS-KEY ( "cross-sectional" )  OR  TITLE-ABS-KEY ( "case control" )  OR  TITLE-ABS-KEY ( "case-control" )  OR  TITLE-ABS-KEY ( "cohort studies" )  OR  TITLE-ABS-KEY ( "cohort study" )  OR  TITLE-ABS-KEY ( allergy )  OR  TITLE-ABS-KEY ( allergies )  OR  TITLE-ABS-KEY ( duck )  OR  TITLE-ABS-KEY ( malaria )  OR  TITLE-ABS-KEY ( parasite* )  OR  TITLE-ABS-KEY ( egg-laying )  OR  TITLE-ABS-KEY ( reproduction )  OR  TITLE-ABS-KEY ( reproductive )  OR  TITLE-ABS-KEY ( cell* )  OR  TITLE-ABS-KEY ( vaccine )  OR  TITLE-ABS-KEY ( allergen )  OR  TITLE-ABS-KEY ( parasitic )  OR  TITLE-ABS-KEY ( vaccination )  OR  TITLE-ABS-KEY ( immunology )  OR  TITLE-ABS-KEY ( immunization )  OR  TITLE-ABS-KEY ( immunise )  OR  TITLE-ABS-KEY ( immunize )  OR  TITLE-ABS-KEY ( "animal models" )  OR  TITLE-ABS-KEY ( "egg yolk agar" )  OR  TITLE-ABS-KEY ( insect* )  OR  TITLE-ABS-KEY ( hen )  OR  TITLE-ABS-KEY ( quail* )  OR  TITLE-ABS-KEY ( hens )  OR  TITLE-ABS-KEY ( chicken* )  OR  TITLE-ABS-KEY ( broiler* )  OR  TITLE-ABS-KEY ( "in vitro" )  OR  TITLE-ABS-KEY ( "in-vitro" )  OR  TITLE-ABS-KEY ( rats )  OR  TITLE-ABS-KEY ( laying )  OR  TITLE-ABS-KEY ( breed* )  OR  TITLE-ABS-KEY ( frog* )  OR  TITLE-ABS-KEY ( worm )  OR  TITLE-ABS-KEY ( fruitworm )  OR  TITLE-ABS-KEY ( semen ) ) ) |
| Web of Science (ISI) |
| (TS=(egg) OR TS=(eggs)) AND (TS=(intervention) OR TS=(intervention*) OR TS=(trial) OR TS=(randomized) OR TS=(blind*) OR TS=(randomised) OR TS=(random) OR TS=(randomly) OR TS=(placebo) OR TS=(assignment) OR TS=(RCT) OR TS=("cross over") OR TS=("cross-over") OR TS=(parallel) OR TS=("body weight") OR TS=("body mass index") OR TS=(BMI) OR TS=("Waist Circumference") OR TS=(WC) OR TS=(WHR) OR TS=(FFM) OR TS=(LBM) OR TS=(FM) OR TS=("Waist-Hip Ratio") OR TS=("lean body mass") OR TS=("fat free mass")) NOT (TS=(mouse) OR TS=(mice) OR TS=(rat) OR TS=(pig) OR TS=(pigs) OR TS=(rabbit*) OR TS=(rooster) OR TS=(roosters) OR TS=(cell) OR TS=(cells) OR TS=(cow) OR TS=(cows) OR TS=(rabbits) OR TS=(hamsters) OR TS=(monkey) OR TS=(monkeys) OR TS=(goats) OR TS=(shrimp) OR TS=(sheep) OR TS=(cats) OR TS=(dogs) OR TS=(crab) OR TS=(fowl) OR TS=(fowls) OR TS=(roots) OR TS=(bacteria) OR TS=(camel) OR TS=(cross sectional) OR TS=(cross-sectional) OR TS=(case control) OR TS=(case-control) OR TS=(cohort studies) OR TS=(cohort study) OR TS=(allergy) OR TS=(allergies) OR TS=(duck) OR TS=(malaria) OR TS=(parasite*) OR TS=(egg-laying) OR TS=(reproduction) OR TS=(reproductive) OR TS=(cell*) OR TS=(vaccine) OR TS=(allergen) OR TS=(parasitic) OR TS=(vaccination) OR TS=(immunology) OR TS=( immunization) OR TS=(immunise) OR TS=(immunize) OR TS=("animal models") OR TS=("egg yolk agar") OR TS=(insect*) OR TS=(hen) OR TS=(quail*) OR TS=(hens) OR TS=(chicken*) OR TS=(broiler*) OR TS=("in vitro") OR TS=("in-vitro") OR TS=(rats) OR TS=(laying) OR TS=(breed*) OR TS=(frog*) OR TS=(worm) OR TS=(fruitworm) OR TS=(semen)) |
